# Large-scale analysis of fact-checked stories on Twitter reveals graded effects of ambiguity and falsehood on information reappearance

**DOI:** 10.1093/pnasnexus/pgaf028

**Published:** 2025-02-19

**Authors:** Julian Kauk, Helene Kreysa, Stefan R Schweinberger

**Affiliations:** Department of General Psychology and Cognitive Neuroscience, Friedrich Schiller University Jena, Am Steiger 3/1, the Free State of Thuringia, 07743 Jena, Germany; Department of General Psychology and Cognitive Neuroscience, Friedrich Schiller University Jena, Am Steiger 3/1, the Free State of Thuringia, 07743 Jena, Germany; Department of General Psychology and Cognitive Neuroscience, Friedrich Schiller University Jena, Am Steiger 3/1, the Free State of Thuringia, 07743 Jena, Germany; Michael Stifel Center Jena for Data-Driven & Simulation Science, Friedrich Schiller University Jena, Leutragraben 1, the Free State of Thuringia, 07743 Jena, Germany; German Center for Mental Health (DZPG), Site Jena-Magdeburg-Halle, Philosophenweg 3, the Free State of Thuringia, 07743 Jena, Germany

**Keywords:** misinformation, fake news, Twitter/X, peak detection, time series analysis

## Abstract

Misinformation disrupts our information ecosystem, adversely affecting individuals and straining social cohesion and democracy. Understanding what causes online (mis)information to (re)appear is crucial for fortifying our information ecosystem. We analyzed a large-scale Twitter (now “X”) dataset of about 2 million tweets across 123 fact-checked stories. Previous research suggested a falsehood effect (false information reappears more frequently) and an ambiguity effect (ambiguous information reappears more frequently). However, robust indicators for their existence remain elusive. Using polynomial statistical modeling, we compared a falsehood model, an ambiguity model, and a dual effect model. The data supported the dual effect model (13.76 times as likely as a null model), indicating both ambiguity and falsehood promote information reappearance. However, evidence for ambiguity was stronger: the ambiguity model was 6.6 times as likely as the falsehood model. Various control checks affirmed the ambiguity effect, while the falsehood effect was less stable. Nonetheless, the best-fitting model explained <7% of the variance, indicating that (i) the dynamics of online (mis)information are complex and (ii) falsehood effects may play a smaller role than previous research has suggested. These findings underscore the importance of understanding the dynamics of online (mis)information, though our focus on fact-checked stories may limit the generalizability to the full spectrum of information shared online. Even so, our results can inform policymakers, journalists, social media platforms, and the public in building a more resilient information environment, while also opening new avenues for research, including source credibility, cross-platform applicability, and psychological factors.

**Significance Statement**

Our study sheds light on the intricate dynamics of (mis)information reappearance on social media, demonstrating that both ambiguity and falsehood significantly contribute to the recurrence of information online. Through the analysis of a large-scale Twitter dataset, we discovered that ambiguity is a more potent predictor of reappearance than falsehood. Nonetheless, our models accounted for only a small fraction of the variance, highlighting the complex and multifaceted nature of online (mis)information dynamics. Despite specific limitations, these findings are pivotal for crafting strategies to strengthen the resilience of our information ecosystem, providing essential guidance for policymakers, journalists, and social media platforms in their efforts to combat misinformation.

## Introduction

The spread of online misinformation has become a significant -global concern, disrupting our information ecosystem (1, 2). We define misinformation as any false or misleading information (3, 4). The effects of misinformation are complex and not fully understood (5), ranging from adverse individual impacts (e.g. reduced adherence to health measures; see Ref. (6)) to broader societal issues, including the erosion of social cohesion and democracy, decreased trust in politicians and the press, and weakened support for climate change mitigation (1, 7–9).

Both academia and the public have made substantial efforts to understand and counteract misinformation (see, e.g. (10–15)). However, the field still faces significant challenges (see Refs. (1, 5, 16, 17)). These challenges include establishing a consistent conceptual framework, developing standardized methods for data collection, and determining the actual prevalence and impact of misinformation.

The key role of social media in amplifying misinformation is widely recognized among scholars (see, e.g. (1, 18–20)). Research focuses on how misinformation spreads on social media and how this differs from the spread of credible information. Understanding and predicting the dynamics of online (mis)information, particularly through the study of information cascades, has attracted significant academic attention (see, e.g. (21–24)); for a survey, see Ref. (25). Significant contributions have enhanced our understanding of the dynamics of false and true information cascades. Vosoughi et al. (15), using a large-scale Twitter dataset, found that false news cascades tend to exhibit greater “fitness” in terms of depth, size, and velocity of spread, likely due to perceived novelty. Zhang et al. (26) examined the diffusion of conspiracy- and science-related cascades on Reddit, finding similar patterns to Vosoughi et al. (15), indicating cross-platform validity.

Epidemic models, originally developed to model the spread of infectious diseases, have significantly influenced research on the spread of online (mis)information. These models are frequently used to characterize the overall time series of (mis)information spread and describe cascade diffusion using graph theory (see, e.g. (11, 27, 28)); see also Refs. (29–31). Generally, epidemic models (i) divide a population into different compartments (in the SIR model, individuals are either susceptible [S], infected [I], or recovered [R] from believing a certain piece of information) and (ii) define the transitions between compartments. Various complex models have been proposed to describe the spread of online (mis)information (see Refs. (31, 32)), including nonepidemic models like threshold models (33) and cascading models (34).

However, simple epidemic models, such as the classic SIR model, and other diffusion models often fail to capture the full lifecycle of (mis)information. This is evident when analyzing time series data from social media, which often show numerous erratic peaks over time (cf. (35–37)). These fluctuations are frequently due to exogenous influences affecting the diffusion of (mis)information. While existing models describe the endogenous topology of networks well, they often overlook exogenous variables that modulate (mis)information dynamics. This gap has been noted by Raponi et al. (31), who stated that “propagation seems to be characterized also by factors that are not endogenous to the graph (data) under analysis” (p. 29). Consequently, many studies neglect the important characteristic of how information evolves over longer periods.

A related research question with significant implications for modeling the spread of online (mis)information is the extent to which misinformation reappears after its initial emergence, and whether this tendency differs from that of more credible information. Answering this question is crucial, as cognitive psychology suggests that the repetitive presentation of (false) information can increase its believability, known as the illusory truth effect (see, e.g. (38, 39). Moreover, a differential temporal pattern of (re)appearance could help in detecting misinformation.

Shin et al. (36) studied the temporal dynamics of false and true rumors on Twitter and found that false rumors tended to resurface, while true rumors did not. Specifically, they observed that true rumors appeared only once, while false rumors reemerged an average of 3.31 times. They also noted that tweet activity was less concentrated on a single day for false rumors (18.57%) compared to true rumors (49.58%), supporting the findings of multiple peaks. However, it is important to acknowledge the limitations of this study. The relatively small and selective sample of 17 rumors (4 true, 13 false) focused on the (pre)-election period of the 2012 U.S. presidential election, primarily centered around Barack Obama and Mitt Romney. Consequently, the generalizability of conclusions drawn from these data may be limited, emphasizing the need for more robust evidence in understanding the relationship between information credibility and temporal dynamics.

A further factor which may play a crucial role in the reappearance of information is ambiguity, as individuals persistently seek confirmation under uncertainty. We here define ambiguity in information as content whose truthfulness is uncertain or indeterminate, often involving mixed, partial, or conflicting truth claims (see also Refs. (40, 41). In this regard, Mitra et al. (40) explored the temporal dynamics of highly and less credible events on Twitter using the CREDBANK dataset (see Ref. (35)). Although less credible events reappeared more frequently, the effect sizes were smaller than those reported by Shin et al. (36). The authors primarily attributed these findings to uncertainty, as their dataset virtually lacked false events (only one event was rated as inaccurate), but largely contained “uncertain,” “probably accurate,” or “certainly accurate” events. The absence of false events limits the generalizability of their findings, as they cannot attribute their findings to ambiguity or falsehood. More causal evidence for the role of ambiguous information is provided by Allen et al. (42), who investigated whether outright misinformation or factually accurate but misleading content was more effective in driving COVID-19 vaccine hesitancy. They found that “gray area” content—factually accurate yet deceptive—was 46 times more influential in fostering vaccine hesitancy than clear misinformation. This underscores the critical importance of addressing such ambiguous content, which often remains unflagged on social media, but may poses a substantial threat to public health and opinion making.

### Research objectives

This work aims to deepen our understanding of the long-term dynamics of online (mis)information through a large-scale analysis of fact-checked stories on Twitter. Data from fact-checking organizations such as Snopes and PolitiFact has been widely used in the literature (see, e.g. (15, 36, 43, 44)). While these organizations often show high agreement when assessing the same story (see Ref. (45)), this approach is not without limitations. Selection biases and resource constraints can influence which stories are fact-checked, raising concerns about the representativeness of the fact-checked dataset (see Refs. (46, 47)). Nevertheless, fact-checking remains a powerful tool for studying online (mis)information. Leveraging its strengths, this work aims to address the following research questions:

1. *Is there an effect of falsehood on the reappearance of online information?* Initial exploratory results suggest false information tends to reappear more frequently than true information (see Ref. (36)), but conclusive evidence is needed.
2. *Is there an effect of ambiguity on the reappearance of online information?* There is preliminary evidence that ambiguous information tends to reappear more frequently than unambiguous information (see Ref. (40)).
3. *If both effects are replicable, which effect is more prominent?* No prior research has simultaneously examined falsehood and ambiguity effects, leaving a gap in understanding their relative importance.

## Methods

### Data

We utilized the adaptive community-response (ACR) method, as presented by Kauk et al. (17), to compile our Twitter dataset. The ACR method leverages data from fact-checking sources to automatically create queries for retrieving tweets associated with specific (misinformation) stories. This method combines keywords from fact-checking sources and adapts to Twitter’s syntax by including Twitter-specific terms relevant to the story. The ACR method is designed to perform only queries that exhibit sufficient precision while maximizing recall for a particular story.

Each story in our dataset was verified by one of two major fact-checking sources, Snopes and PolitiFact (48, 49). These sources primarily validate stories related to the United States but also address global topics. Relying on fact-checkers’ verdicts is a standard practice in the field (cf. (15, 18, 36, 50–52)), allowing researchers to accurately assess information credibility. Fact-checking organizations typically use an ordinal scale to rate story credibility, usually ranging from “highly false” to “completely true.” Consistent with prior research (cf. (15, 35)), we employed a five-level Likert scale to represent the credibility of the stories (see Table 1). Basic information for each story (including story claim, fact-checking source, query, number of tweets, onset, and end) is available as a .csv file on OSF.

**Table 1.** Mapping of the textual ratings of the fact-checking pages to the five-level Likert scale.

Fact-checking source	− 2	− 1	0	1	2
	False	Mostly false	Mixed	Mostly true	True
Snopes	“False” “Scam”	“Mostly False”	“Mixture”	“Mostly True”	“True”
PolitiFact	“Pants on Fire” “False”	“Mostly False”	“Half True”	“Mostly True”	“True”

While we categorize “False” and “True” stories as misinformation and true information, respectively, we classify the intermediate categories as ambiguous information. Ambiguous information, in line with our definition provided in the introduction, refers to content rated by fact-checkers as “Mostly false,” “Mixed,” or “Mostly true,” indicating that the veracity is not clear-cut and allows for multiple interpretations.

#### Data cleaning and preparation

We conducted several steps to enhance data quality and prepare it for final analyses. A crucial step involved excluding tweets that were (i) irrelevant or (ii) contrary to the specific story. This was accomplished by combining basic natural language processing (NLP) tools with advanced transformer-based models, which have notably advanced NLP performance recently (cf. (53)).

Initially, we cleaned our tweets (we only considered original tweets and their retweets) by removing unwanted characters. Specifically, URLs and various irregular characters were eliminated, allowing only alphanumeric characters ([A-Za-z0-9]) and specific punctuation characters ([?!.,’]). All text was converted to lowercase. Mentions (@) and hashtags (#) were kept, though the symbols @ and # were removed, as mentions and hashtags often hold crucial context in tweets. To filter out irrelevant tweets, we utilized a sentence-transformers model (see Refs. (54, 55)) to measure semantic similarity between tweets and story claims. Specifically, we calculated the cosine similarity $S_{c}(t,c)\in [$-1,1] between each tweet *t* and its corresponding claim *c*. This technique is prevalent in the literature (see, e.g. (15, 17, 36, 56)) and enables researchers to exclude irrelevant tweets based on a text similarity threshold. Generally, two texts are considered to have a certain degree of similarity if $S_{c}(t,c)\geq 0.5$ (cf. (15, 36, 57)), while two texts are nearly identical if $S_{c}(t,c)\geq 0.9$.

The optimal threshold for balancing recall and precision can vary depending on the NLP tool, typically lying in an intermediate range due to the precision-recall tradeoff (17). Considering these factors, we predefined a text similarity threshold of 0.7, excluding tweets below this mark. Additionally, we excluded tweets containing (i) words that indicate doubt (e.g. “misinformation,” “fact-check,” “fake news”) or (ii) links to our fact-checking sources (i.e. Snopes or PolitiFact). Furthermore, we used a pretrained RoBERTa-Large natural language inference (NLI) model (58) to filter out any remaining tweets expressing doubt about the claim. NLI is an NLP task that determines the (logical) relationship between two sentences; specifically, it identifies whether a tweet logically contradicts a claim.

Finally, to enhance the robustness of our response variables, we excluded 1% of the tweets from each story’s distribution tails based on timestamps. This ensured that the story’s onset and end were estimated reliably, as the ACR method was designed to search for tweets over relatively broad timeframes. We only included stories with at least 3,000 tweets, aligning with established thresholds for the minimum number of tweets (see, e.g. (17, 36)).

### Response variables

We used the same measures as Shin et al. (36, p. 281) to characterize the temporal dynamics of the stories. We will introduce the two measures, peak frequency and burstiness, in the upcoming sections.

####  

##### Peak frequency

This measure is based on a simple peak-finding algorithm capable of identifying local maxima by comparing neighboring data points. We applied two criteria to ensure that the peaks represented meaningful entities. A minimal height of 10% of the maximal value of the time series and a minimum distance of 7 days between peaks were used to ensure that peaks were significant, truly reflecting a reoccurrence of the story.

Formally, we consider a time series *X*, as given by


(1) $$X=(x_{1},x_{2},\ldots ,x_{n}),$$

where $x_{i}$ represents the number of tweets posted on day *i* and *n* is the total number of days. A data point


(2) $$x_{i}\;\mathrm{is}\;a\;\mathrm{peak}\;\mathrm{iff}=\left\{ \begin{array}{c} x_{i}>x_{i+k},\\ x_{i}>x_{i-k}\;\mathrm{and}\\ x_{i}\geq 0.1\times \max(X), \end{array}\right.$$

where k={x∣x∈Z,1≤x≤7}. All data points that meet these criteria belong to the peaks set, denoted Peaks(X). The peak frequency is therefore given by


(3) Peakfrequency=|Peaks(X)|.

The peaks set (Peaks(X)) and its cardinality (Peakfrequency) were determined using the function Find_peaks from the Scipy library (version 1.10.1).

##### Burstiness

This measure reflects the concentration of tweets, calculated as the ratio of the maximum deflection in the time series to its total sum. Burstiness is therefore given by:


(4) $$\mathrm{Burstiness}=\;\frac{\max(X)}{\sum_{i=1}^{n}X_{i}}.$$

A Burstiness approaching 1 indicates a high concentration of tweets on a single day, while lower values indicate more dispersed tweet activity. Burstiness should exhibit a high negative correlation to peak frequency, as stories with multiple peaks should also exhibit less concentration.

### Modeling

We used two types of regression models within the generalized linear model (GLM) framework to analyze our response variables. We modeled peak frequency using zero-truncated negative binomial regression (59), a statistical technique designed for count data without zero counts and capable of accounting for overdispersion (i.e. variance greater than the mean). We used the r package Countreg (version 0.3-0) for maximum likelihood estimation of the parameters. The link function is defined as:


(5) $$\log(\mu_{i})=\eta_{i},$$

where $\mu_{i}$ is the expected value of the peak frequency for story *i* and $\eta_{i}$ is the linear predictor.

We employed beta regression (60) to model burstiness, which is well-suited for continuous data constrained within the interval (0, 1). For this, we used the r package Betareg (version 3.1-4) for maximum likelihood estimation of the parameters. The link function used is:


(6) $$\mathrm{logit}(\mu_{i})=\eta_{i},$$

where $\mu_{i}$ is the expected value of the burstiness for story *i* and $\eta_{i}$ is again the linear predictor.

In this study, we investigate three model structures: linear, quadratic without a linear term, and quadratic with a linear term. These structures are applied using both zero-truncated negative binomial regression and beta regression. The detailed model structures will be explained in the following paragraphs.

####  

##### Falsehood model

We tested the relationship between the rating of the stories (see Table 1) and the response variable (peak frequency or burstiness) via a linear model stricture. We assumed linear relationships between information credibility and our response variables, corresponding to the falsehood effect reported by Shin et al. (36). This model assumes no effect of ambiguity on the response variables, meaning that the variance of our outcome variables is solely explainable by the falsehood effect.

The model is given by:


(7) $$\eta_{i}=\;\beta_{0}+\beta_{1}x_{i},$$

where $\eta_{i}$ is the linear predictor of the response variable (either Peakfrequency or Burstiness), $x_{i}$ is the fact-checking rating (see Table 1), $\beta_{0}$ is the intercept, and $\beta_{1}$ is the slope.

##### Ambiguity model

We used a constrained quadratic model structures without a linear term to model the relationship between the rating of the stories and our outcome variables. This model resembles the proposed ambiguity effect reported by Mitra et al. (40), where mixed information (exhibiting maximal ambiguity) should have the highest number of peaks. This model assumes an effect of ambiguity only, with no effect of falsehood.

The model is given by:


(8) $$\eta_{i}=\;\beta_{0}+\beta_{2}x_{i}^{2},$$

where $\eta_{i}$ is the linear predictor of the response variable (either Peakfrequency or Burstiness), $x_{i}$ is the fact-checking rating (see Table 1), $\beta_{0}$ is the intercept, and $\beta_{2}$ is the quadratic coefficient.

##### Dual effect model

Finally, we employed an unconstrained quadratic model structure to examine the relationship between the rating of stories and our response variables, assuming modulation by both falsehood and ambiguity (see Refs. (36, 40)). This model incorporates both a linear term (falsehood effect) and a quadratic term (ambiguity effect).

The model is represented as:


(9) $$\eta_{i}=\;\beta_{0}+\beta_{1}x_{i}+\beta_{2}x_{i}^{2},$$

where $\eta_{i}$, $x_{i}$, and $\beta_{0}$ retain their meanings from the linear model, and $\beta_{1}$ and $\beta_{2}$ are the linear and quadratic coefficients, respectively.

##### Model selection

We used (pseudo) coefficients of determination $R^{2}$ and the Akaike information criterion (AIC; see Ref. (61)) to estimate our models’ goodness-of-fit. While $R^{2}$ reflects the proportion of variance explained, AIC accounts for model complexity. We compared models to select the one best explaining the observed data. For AIC differences, we followed the rules of thumb proposed by Burnham and Anderson (62), considering ΔAIC≤2 as indicating no support for the model with the lower AIC. We also report the relative likelihood (RL) of models $M_{1}$ and $M_{2}$, calculated as (see Ref. (63))


(10) $$\mathrm{RL}=\exp\left(\frac{\mathrm{AIC}(M_{1})-\mathrm{AIC}(M_{2})}{2}\right).$$

Thus, RL reflects the relative likelihood of $M_{1}$ compared to $M_{2}$. When models had different numbers of parameters, we used likelihood ratio tests to compare goodness-of-fit, providing inferential evidence on whether a model explains the data more accurately considering parameter differences. Furthermore, we provide numerical values for the corrected Akaike Information Criterion (AICc), which adjusts for small sample sizes (see Ref. (63)), as well as the Bayesian Information Criterion (BIC; see (61)).

Table 2 summarizes the model structures considered in the present study, including a null model without falsehood and ambiguity effects.

**Table 2.** Model structures under considerations in the present study and the respective effects they are testing.

Model	Equation	Effect
Intercept	$\eta_{i}=\beta_{0}$	No effect
Linear	$\eta_{i}=\beta_{0}+\beta_{1}x_{i}$	Falsehood effect
Quadratic (constr.)	$\eta_{i}=\beta_{0}+\beta_{2}x_{i}^{2}$	Ambiguity effect
Quadratic (unconst.)	$\eta_{i}=\beta_{0}+\beta_{1}x_{i}+\beta_{2}x_{i}^{2}$	Dual effect of falsehood and ambiguity

## Results

Note that all model outputs and scripts are available on OSF. All models converged.

### Tweet sample

Overall, we recorded 3,110,773 tweets using the ACR method, showing a text similarity of 0.7 or higher with the corresponding story claim. We excluded 291,728 (9.38%) tweets containing words expressing doubt about a story, and 544,757 (17.51%) tweets containing links to our fact-checking sources. Furthermore, using the NLI model, we excluded 172,530 (5.55%) tweets classified as “contradictory” to the story claim. Lastly, we excluded 1% of the tweets from each story’s distribution tails to enhance the robustness of the response variables and story onset and end estimations.

In total, 123 stories (false: 92, mixed: 9, and true: 22) surpassed the threshold of 3,000 tweets, totaling 1,902,842 tweets. On average, each story had 15,470.26 tweets. Our tweets spanned from February 2016 to August 2022, with most tweets recorded around June 2020 (IQR: October 2019–December 2020), indicating that most were posted within the past decade. Story duration varied, with a median duration of 145 days (IQR: 26–314 days), suitable for time series analysis. No systematic differences were found in the median duration of false, mixed, and true stories, confirmed by a Kruskal–Wallis *H*-test (H(4)=7.18,P=0.127). Similarly, no meaningful differences were found in their median onsets (H(4)=7.06,P=0.133).

Our dataset encompasses various domains and topics, including elections, climate change, COVID-19, economy, and Ukraine, among others (see the word cloud in the Supplementary file 1, Fig. 1). A summary table detailing all 123 stories is available on OSF.

**Fig. 1.**
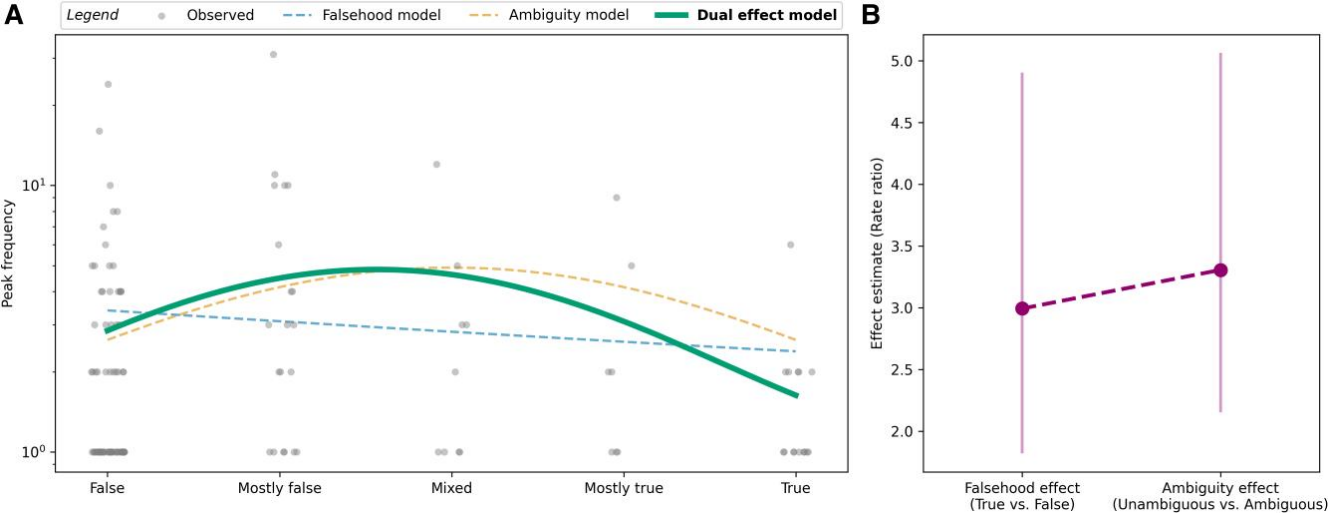
Falsehood, ambiguity, and dual effect models for peak frequency. A) Relationship between rating and peak frequency, where dashed curves represent the fits of the inferior models, and the solid line represents the fit of the superior model. B) Effect estimates (rate ratios) for the falsehood and ambiguity effects, with error bars indicating standard errors.

### Peak frequency

Over all stories, peak frequency ranged from 1 to 31 with a mean of 3.12 (SD: 4.2) and a median of 2.0 (IQR: 1.0−−3.5). Sixty (48.78%) stories exhibited only one peak, indicating a right skewed-distribution of the peak frequency (skewness: 3.97; see also the additional distribution figure in the Supplementary material). The main results of the zero-truncated negative binomial regression models are shown in Table 3.

**Table 3.** Main results of the zero-truncated negative binomial regression models for peak frequency.

	Coefficient			Model diagnostic					Relative likelihood			
Model	$\beta_{0}$	$\beta_{1}$	$\beta_{2}$	$R_{CS}^{2}$	log(L)	AIC	AICc	BIC	Null	Falseh.	Ambig.	Dual
Null	−8.72	–	–	0	−226.6	457.19	457.29	462.81	–	1.61	$0.24^{*}$	$0.07^{**}$
Falsehood	−8.79	−0.16	–	$2.31\times 10^{-3}$	−226.07	458.14	458.24	466.58	0.62	–	0.15	$0.05^{**}$
Ambiguity	−7.99	–	$-0.27^{*}$	0.011	−224.18	454.37	454.47	462.8	$4.1^{*}$	6.6	–	$0.3^{*}$
Dual effect	−7.15	$-0.31^{*}$	$-0.36^{**}$	0.02	−221.97	451.95	452.04	463.19	$13.76^{**}$	$22.16^{**}$	$3.36^{*}$	–

*Note.* *P<0.05 , **P<0.01, ***P<0.001. $R_{\mathrm{CS}}^{2}$ reflects pseudo-$R^{2}$ according to Cox and Snell. Asterisks behind the relative likelihoods reflect results of the likelihood ratio tests.

####  

##### Falsehood model

Initially, we examined whether the rating linearly predicted peak frequency, resembling a falsehood effect. As anticipated, the model indicated a negative relationship between rating and peak frequency (see Fig. 1A). However, the linear coefficient was not significantly different from zero ($\beta_{1}=-0.16,P=0.287$), suggesting that the model did not explain a significant amount of variability in the data compared to the null model. This superiority of the null model was confirmed by the AICs, with the null model exhibiting a lower AIC (457.19) than the falsehood model (458.14). The AIC difference was 0.95, indicating that the null model is 1.61 times as likely as the falsehood model. A likelihood ratio test confirmed that the observed data is equally likely under both models ($\chi^{2}(1)=1.05,P=0.306$).

##### Ambiguity model.

Subsequently, we tested whether the rating predicted peak frequency in a quadratic fashion, indicating an ambiguity effect. As expected, the model predicted an inverted U-shaped curve (see Fig. 1A), supported by a significant negative quadratic coefficient ($\beta_{2}=-0.27,P=0.038$), indicating that ambiguity modulated peak frequency. The AIC of the ambiguity model (454.37) confirmed its superiority over both the null model (ΔAIC=2.82) and the falsehood model (ΔAIC=3.78), implying that the ambiguity model was 4.1 and 6.6 times as likely as the null and falsehood models, respectively. A likelihood ratio test confirmed that the observed data are more likely under the ambiguity model than under the null model ($\chi^{2}(1)=4.82,P=0.028$).

##### Dual effect model

Finally, we evaluated whether a dual effect model, incorporating both a linear term (falsehood effect) and a quadratic term (ambiguity effect), could predict peak frequency (see Fig. 1A). Consistent with the ambiguity model, we found a significant negative quadratic coefficient ($\beta_{2}=-0.36,P=6.59\times 10^{-3}$), confirming that ambiguity modulated peak frequency. Importantly, the dual effect model also showed a significant negative linear coefficient ($\beta_{1}=-0.31,P=0.024$), reflecting a falsehood effect, where more credible information tends to have fewer peaks. Interestingly, the falsehood effect emerged only when ambiguity was modeled with a quadratic term. We presume the stronger ambiguity effect overshadowed the falsehood effect, explaining the initially observed insignificance of the falsehood model.

The AIC of the dual effect model (451.95) confirmed its superiority over all previously reported models, with all AIC differences exceeding 2 (5.24, 6.2, and 2.42 for the intercept, falsehood, and ambiguity models, respectively). The dual effect model was 13.76, 22.16, and 3.36 times as likely as the intercept, falsehood, and ambiguity models, respectively. Likelihood ratio tests confirmed the higher likelihood of the dual effect model over all other models (P<0.05 for all comparisons). The dual effect model showed the smallest improvements relative to the ambiguity model, indicated by relatively low AIC differences and improvements in likelihood, confirming previous observations of the dominance of ambiguity over falsehood effects.

The relatively greater impact of ambiguity is also illustrated in Fig. 1B, where each effect is treated as a dichotomous variable, reflecting differences between false (rating: −2 and −1) and true stories (rating: 1 and 2), or between unambiguous (rating: −2 and 2) and ambiguous (rating: −1, 0, and 1) stories.^1^ We report rate ratios as effect estimates, showing the multiplicative increase in peak frequency associated with each effect. Despite substantial uncertainty in the effect estimates, the estimates were higher for ambiguity (3.31) than for the falsehood effect (2.99), suggesting ambiguity is a more important predictor.

### Burstiness

Over all stories, burstiness ranged from 0.03 to 0.92 with a mean of 0.43 (SD: 0.22) and a median of 0.39 (IQR: 0.26−−0.61). The distribution of burstiness was only moderately skewed (skewness: 0.24). As predicted, we found peak frequency and burstiness to be highly correlated (Spearman’s ρ(121)=−0.791,P≈0). Additional distribution and correlation figures are provided in the Supplementary material. The main results of the beta regression models are shown in Table 4.

**Table 4.** Main results of the beta regression models for burstiness.

	Coefficient			Model diagnostic					Relative likelihood			
Model	$\beta_{0}$	$\beta_{1}$	$\beta_{2}$	$R_{CS}^{2}$	log(L)	AIC	AICc	BIC	Null	Falseh.	Ambig.	Dual
Null	$-0.29^{***}$	–	–	–	19.29	−34.58	−34.49	−28.96	−	1.85	$0.25^{**}$	$0.17^{*}$
Falsehood	$-0.24^{*}$	0.05	–	$6.22\times 10^{-3}$	19.68	−33.35	−33.25	−24.91	0.54	–	0.14	$0.09^{**}$
Ambiguity	$-0.64^{***}$	–	$0.11^{*}$	.039	22.67	−37.34	−37.24	−28.9	$3.97^{**}$	7.36	–	0.68
Dual effect	$-0.63^{***}$	0.1	$0.14^{**}$	.061	23.06	−38.13	−38.03	−26.88	$5.88^{*}$	$10.89^{**}$	1.48	–

*Note.* *P<0.05 , **P<0.01, ***P<0.001. $R_{\mathrm{McF}}^{2}$ reflects pseudo-$R^{2}$ according to McFadden. Asterisks behind the relative likelihoods reflect results of the likelihood ratio tests.

####  

##### Falsehood model

**Fig. 2.**
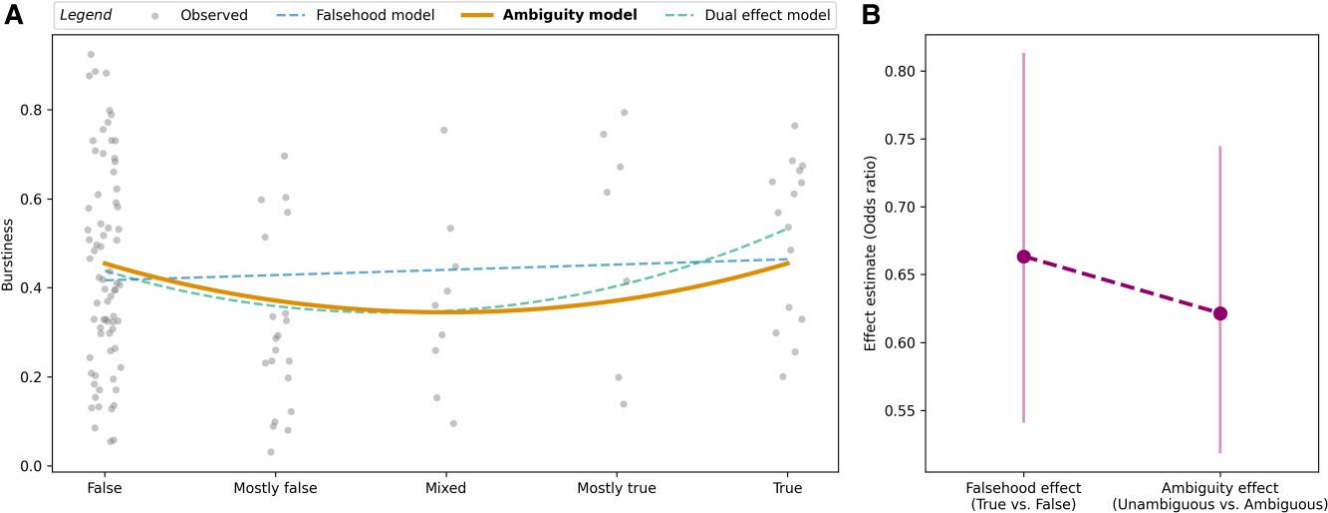
Falsehood, ambiguity, and dual effect models for burstiness. A) Relationship between rating and burstiness, where dashed curves represent the fits of the inferior models, and the solid line represents the fit of the superior model. B) Effect estimates (odds ratios) for the falsehood and ambiguity effects, with error bars indicating standard errors.

The falsehood model indicated a positive relationship between rating and burstiness (see Fig. 2A). However, similar to our findings for peak frequency, the linear coefficient was not significantly different from zero ($\beta_{1}=0.05,P=0.376$), suggesting that the model did not explain a significant amount of variability compared to the null model. This was confirmed by the AICs, where the null model exhibited a lower AIC (−34.58) than the falsehood model (−33.35), corresponding to an AIC difference of 1.23. This indicates that the null model is 1.85 times as likely as the falsehood model. A likelihood ratio test confirmed that the observed data is equally likely under both models ($\chi^{2}(1)=0.77,P=0.381$).

##### Ambiguity model

The ambiguity model predicted a U-shaped curve (see Fig. 2A), supported by a significant negative quadratic coefficient ($\beta_{2}=0.11,P=0.029$), consistent with previous findings regarding ambiguity and peak frequency. The AIC of the ambiguity model (−37.34) confirmed its superiority over both the null (ΔAIC=2.76) and the falsehood model (ΔAIC=3.99). This implies that the ambiguity model was 3.97 and 7.36 times as likely as the null and falsehood models, respectively. A likelihood ratio test confirmed that the observed data is more likely under the ambiguity model than under the null model ($\chi^{2}(1)=6.75,P=9.35\times 10^{-3}$).

##### Dual effect model

In the dual effect model, we found a significant positive quadratic coefficient ($\beta_{2}=0.14,P=9.18\times 10^{-3}$), confirming that ambiguity modulated burstiness. However, unlike our results for peak frequency, the dual effect model did not indicate a significant positive linear coefficient ($\beta_{1}=0.1,P=0.092$), reflecting a falsehood effect. This means that more credible information does not significantly correlate with less burstiness.

The AIC of the dual effect model (−38.13) confirmed its superiority over the null (ΔAIC=3.54) and falsehood model (ΔAIC=4.78), but not over the ambiguity model (ΔAIC=0.79), as the difference was smaller than 2. The dual effect model was 5.88 and 10.89 times as likely as the null and falsehood models, respectively, but only slightly more likely (relative likelihood: 1.48) as the ambiguity model. Likelihood ratio tests confirmed the higher likelihood of the dual effect model over the null and falsehood models (P<.05 for both), while no such superiority was observed over the ambiguity model ($\chi^{2}(1)=0.79,P=0.374$).

The relatively greater effects of ambiguity are again expressed in Fig. 2B, where each effect is treated as a dichotomous variable. The effect estimates (odds ratios) were higher for ambiguity (0.62) than for the falsehood effect (0.66), indicating graduation of both effects.

### Control analyses

We conducted a set of control analyses to ensure the robustness of our main analysis. Here, we present the main results of the control analyses, with further information provided in the Supplementary material.

####  

##### Poisson models for peak frequency

Zero-truncated negative binomial regression models address overdispersion in the data using an additional parameter. Poisson regression models do not include this parameter, making them more parsimonious. Model comparisons confirmed that zero-truncated negative binomial regression models were appropriate, as all Poisson models showed signs of overdispersion, confirmed by likelihood ratio tests (P<0.001 for all models).

##### Control models: Cubic and saturated models

To validate the robustness of our superior models (the dual effect model for peak frequency and the ambiguity model for burstiness), we used two overparameterized models as controls: a cubic model (including linear, quadratic, and cubic terms) and a saturated model (treating the fact-checking rating as a categorical variable). Our superior models outperformed the cubic and saturated models for both response variables, as confirmed by likelihood ratio tests and AIC differences.

##### One-inflation check for peak frequency

We also checked for one-inflation of the peak frequency, which would indicate an overrepresentation of one counts in the data. We found no evidence of one-inflation, as confirmed by likelihood ratio tests.

##### Orthogonalization of linear and quadratic predictors

Predictors within a polynomial regression can be correlated, leading to biased estimates of the regression coefficients. We orthogonalized our polynomials, resulting in uncorrelated linear and quadratic predictors, which allowed us to better distinguish both effects. The orthogonal polynomial regression modeling confirmed the superiority of ambiguity effects over falsehood effects. While predicting more significant ambiguity effects, the significance of the linear coefficients declined. These findings confirm the prominence of ambiguity effects and demonstrate that falsehood effects are less stable than previously reported in the literature.

##### Sensitivity analysis for the text similarity threshold

To ensure that the observed effects were not dependent on a specific threshold, we tested the models across a wide range of threshold values (from 0.5 to 0.9). For peak frequency, the linear effect of story credibility showed limited reproducibility, suggesting it was relatively unstable. In contrast, the quadratic effect, which captures ambiguity, was much more consistent across thresholds. Similarly, for burstiness, the quadratic model revealed a stable ambiguity effect, indicating that the results were robust across different threshold choices.

##### Sensitivity analysis for the minimum tweet count per story

We performed a sensitivity analysis to assess whether our findings held across different thresholds for the minimum number of tweets per story. For peak frequency, the linear effect of story credibility was found to be rather weak and inconsistent, while the quadratic effect showed much greater stability, pointing to a stronger ambiguity effect. Similarly, for burstiness, the quadratic effect remained stable across different thresholds, suggesting that the ambiguity effect is robust and less influenced by the minimum tweet count per story.

##### Sensitivity analysis for the peak detection parameters

We employed the peak-finding algorithm proposed by Shin et al. (36), which detects local maxima by comparing neighboring data points. In line with Shin et al. (36), we applied a minimum peak height of 10% of the maximum deflection in the time series and enforced a minimum interval of 7 days between peaks to ensure that each peak represented a meaningful story reappearance. To assess the robustness of these criteria, we performed a control analysis by systematically varying both the height and interval thresholds. The analysis confirmed the robustness of the ambiguity effect across a broad parameter space, while the linear effect of story credibility was more variable.

##### Control model incorporating tweet- and user-level covariates

We conducted a control analysis by adjusting the resolution of our model from the story level to the peak level and incorporating relevant covariates. Using a mixed ordinal regression, we examined the effects of tweet and user characteristics (tweet length, hashtags, mentions, text similarity with the original claim, negative sentiment, follower count, and account verification) on peak frequency. The results showed a robust ambiguity effect on story reappearance and significant effects for text similarity and negative sentiment, while the falsehood effect was not significant anymore.

##### Control analysis excluding “Scam” and “Pants on Fire” categories

To address potential inconsistencies with the 5-point Likert scale, we conducted a control analysis excluding the “Scam” and “Pants on Fire” categories. The results confirmed the robustness of the dual effect model for peak frequency and the ambiguity model for burstiness. For peak frequency, the linear and quadratic coefficients remained significant, and the dual effect model showed the best fit based on AIC values. Similarly, for burstiness, the ambiguity model retained its significance and exhibited the best model fit.

##### Reanalysis of Mitra et al. (40)

We performed a preliminary reanalysis of the data provided by Mitra et al. (35, 40) to determine whether an ambiguity model my also better predict their data. Despite the limitations of their dataset, particularly the virtual absence of inaccurate events, our modeling indicated a slight superiority of the ambiguity over the falsehood model for burstiness. The ambiguity model was 1.4 times as likely as the falsehood model, suggesting that ambiguity may be more effective than falsehood effects in explaining the data.

##### Generalizability of results across fact-checking sources

We tested the dual effect model for peak frequency and the ambiguity model for burstiness separately for Snopes and PolitiFact to evaluate the generalizability of our findings. Both sources showed consistent trends, supporting the presence of ambiguity and falsehood effects, despite differences in their story selection criteria and methods of analysis. These preliminary results suggest that the observed patterns are robust across diverse fact-checking approaches.

##### Temporal stability of false stories: exploring potential effects of Twitter policy changes

Twitter’s moderation policies may have influenced our results by suppressing certain types of misinformation, particularly following major political events. To assess this, we analyzed trends in the peak frequency and burstiness of false stories over time. Using both a temporal median split and generalized additive models (GAMs), we found no evidence of significant linear or nonlinear temporal effects on these variables. While our findings suggest minimal impact of policy changes on the temporal dynamics of false stories in this dataset, future studies with larger datasets could offer greater sensitivity to detect subtle effects.

##### Validating model significance with permutation tests

We employed permutation tests to validate the significance of our models, leveraging their robustness to violations of assumptions and suitability for small datasets. By comparing observed *z*-values to null distributions generated through resampling, we confirmed the robustness of the ambiguity effect across both response variables. However, the falsehood effect proved less stable, with its significance diminished under permutation testing.

## Discussion

In this study, we evaluated the long-term temporal dynamics of a collection of stories, each with a fact-checking rating. This addressed a significant research gap, as longitudinal studies of (mis)information dynamics are rare. We explored the relationship between the credibility of information and its temporal patterns. Consistent with previous research (see Ref. (36)), we observed that false information reappeared more frequently than true information. More importantly, however, this relatively small effect was accompanied by a pronounced ambiguity effect. Specifically, we found that ambiguous information is less concentrated but reappears more often than unambiguous information. Our study contributes to the literature by tracking misinformation stories over an extended period, highlighting important dynamics in the spread of (mis)information.

### Theoretical considerations

The present study establishes the dual effect of ambiguity and falsehood, previously investigated only individually. This dual influence has profound implications for our understanding of the information ecosystem on social media, which will be discussed below. However, our study does not explain why both ambiguous and false information reoccur more frequently than unambiguously true information. Future work should explore the underlying mechanisms.

Regarding the falsehood effect, we suspect that two factors are involved. First, misinformation, when distributed as disinformation (i.e. with intent) (cf. (3)), may be redistributed by certain spreaders to generate additional public attention. Spreaders may be humans or bots, the latter possibly programmed to do so deliberately (see Ref. (64)). This study did not investigate the role of bots in redistributing misinformation; future research should explore which user groups tend to “reawaken” misinformation.

Another possible explanation for the falsehood effect is the emotional language of misinformation. Misinformation often carries higher emotional arousal than true information (cf. (65, 66), which may help it persist longer in the receiver’s mind and generate sustained attention. Disinformation spreaders may intentionally increase emotional arousal to achieve better spread. However, the extent to which this contributes to the falsehood effect remains to be fully explored.

The prominent ambiguity effect could be explained by cognitive factors: Uncertainty about an event or piece of information elicits greater cognitive involvement than certainty, as it makes the environment less predictable (67). As a result, individuals may seek further information to increase predictability, which could explain the tendency of ambiguous information to reappear. This explanation aligns with Mitra et al. (40), who argued that “perhaps the underlying uncertainty of an event is what keeps people yearning for additional information…” (p. 11). In-depth analyses of content changes over time may provide a better understanding of why ambiguous information reappears.

Both effects contributed differently to explain the variance of our response variables. We observed a much more pronounced ambiguity effect, whereas the falsehood effect had a smaller impact. The comparatively limited importance of the falsehood effect may contrast with initial findings by Shin et al. (36), who reported that “most false rumors repeated periodically [11 out of 13], whereas true rumors did not [0 out of 4]” (p. 284). Our data, based on a larger and more heterogeneous dataset, suggest a weaker falsehood effect than their initial report.

### Practical implications

The present study has significant implications for both the scientific community and the public. Politicians, journalists, and social media providers could all benefit from our findings. These groups should be aware of the illusory truth effect, which posits that repetitive presentation of information increases its plausibility. Our findings suggest that false and ambiguous information is more plausibly enhanced compared to unambiguously true information. Malicious actors on social media may exploit this by designing “misinformation attacks” that mix false information with some true aspects, leveraging both falsehood and ambiguity. Our data support this, showing that “mostly false” information had more peaks compared to all other ratings.

These findings have specific implications for the aforementioned groups. Politicians should craft clear public statements to reduce the risk of their statements being repeatedly echoed. Journalists should strive to reduce ambiguity in their reporting and engage in peer-to-peer fact-checking to decrease the prevalence of false information. Social media providers should consider our findings when designing their platforms. They could use the reappearance of information as a signal to detect false and ambiguous content, allowing them to flag such information with warnings, potentially reducing its spread (see, e.g. (28)). Timing is crucial: while novel and unreviewed information may promise high outreach, it often comes at the expense of accuracy and clarity. In the public interest, all groups should act carefully and prudently, prioritizing the accuracy of public discourse over speed.

### Limitations and future work

It is important to note that the effects of both falsehood and ambiguity accounted only for a low proportion of the variance in our response variables, with the best models explaining less than 7%. The dynamics of online (mis)information are complex, and the corresponding time series can appear chaotic. Analyzing online social data requires great care and methodological rigor (see Ref. (68)), as it is generated through a complex interplay of users (humans or bots), platform architecture, and external real-world events. This complexity may explain the low variance explained. Identifying and describing the many potentially relevant factors will be challenging but essential for better predicting the dynamics of online (mis)information across various platforms. In this context, incorporating topic-specific characteristics (e.g. political or health-related content) into the model could be beneficial. This approach may not only increase the variance explained but also help account for potential confounding effects. Future studies with larger sample sizes of stories could further enhance our understanding of what drives information to resurface by accounting for topic information.

Improved peak identification could help reduce noise in the data. The peak detection algorithm proposed by Shin et al. (36) is a good starting point but has weaknesses: its parameter values are chosen arbitrarily, and the results depend on these values. Burstiness, another response variable, may be more robust since it does not depend on parameter values; however, it is only an indirect measure of peak frequency. Future studies could employ more advanced temporal analyses to improve peak detection. Moreover, operationalizing ambiguity at the tweet level represents a promising avenue for future research: In the present study, ambiguity is assessed exclusively through the verdict of a fact-checking source. Incorporating tweet-level measures of ambiguity, such as perplexity or entropy, could enhance our understanding of the specific types of ambiguity that drive the reappearance of information.

This study offers valuable insights into the behavior of online (mis)information while highlighting opportunities for future research to broaden its scope. Expanding beyond fact-checked stories, future work could integrate diverse data sources, such as content from different social media platforms or news domains (see, e.g. (46)). Combining these sources through a mixed-method approach could enable a deeper understanding of the dynamics between low- and high-credibility content. Fact-checked stories, although precise due to rigorous professional evaluation, are potentially subject to selection biases, as organizations may prioritize certain types of stories for verification (46, 47, 69). Meanwhile, analyzing content from broader news domains may capture a wider variety of stories, albeit with lower accuracy since not all items undergo individual scrutiny. Previous research has demonstrated parallels in the trends observed with both approaches (15), suggesting their complementarity. Future studies should delve further into the relationship between these methods to enhance our understanding of the complex ecosystem of online (mis)information.

Future studies should examine the impact of social networking service policy changes on response variables more closely. In this study, data were accessed post hoc via the Twitter API, meaning some tweets or accounts may have been deleted or suspended due to policy changes and were therefore inaccessible during data collection. This issue is particularly relevant to misinformation following major political events, such as the 2016 U.S. presidential election or the January 6, 2021, Capitol attack (see Refs. (70, 71)). Twitter is notably affected by tweet and account deletions, even within the first few days of publication (e.g. (72)). Zubiaga (73) demonstrated that Twitter data availability diminishes over time, with losses that can exceed 30% after 4 years. While some of our stories may have been subject to similar data loss, Zubiaga also found that such losses do not necessarily compromise representativity of the collected data. This is encouraging for our study, but future research should explicitly model the effects of such deletions, considering that they may not be uniformly distributed across different types of information.

Nonetheless, recent limitations on accessing social media data, particularly from platforms like Twitter/X, present serious challenges for this area of research and raise concerns about the future of academic inquiry in this field. Since Elon Musk’s acquisition of Twitter, changes to API pricing and restrictions on data scraping have made it nearly impossible to obtain the necessary data (cf. (74)), hindering ongoing and future studies. In this study, a mixed-method approach could have strengthened the relevance of our conclusions. However, we were restricted to analyzing a limited number of 123 fact-checked stories, leaving the examination of news domain dynamics incomplete. Like many others, we lost access to the Twitter API following Musk’s takeover, which has limited our capacity to fully explore the dynamics of online (mis)information. We call on social media platforms, policymakers, and other stakeholders to facilitate greater public access to social media data for researchers. This is essential to addressing critical issues in the online space, such as misinformation, hate speech, polarization, and censorship.

## Conclusion

In this study, we analyzed a large-scale Twitter dataset and uncovered a dual effect of ambiguity and falsehood on the tendency of information to reappear. While initial evidence existed for both effects, their simultaneous presence had not been confirmed before. Our work addressed this gap and provided insights into their relative importance, revealing that the ambiguity effect is considerably more pronounced than the falsehood effect. These findings can guide politicians, journalists, and social media providers in designing information architectures that promote a more resilient online information ecosystem.

## Supplementary Material

pgaf028_Supplementary_Data

## Data Availability

The data and code underlying this article are available in the Open Science Framework (OSF) at https://osf.io/863cu/?view_only=9e463738a64f431981236a9708f2dd45.

## Footnotes

1 The other respective effect is still considered as a continuous variable in the regression model.
