# Supplementary material for: Large-scale analysis of fact-checked stories on Twitter reveals graded effects of ambiguity and falsehood on information reappearance
Source: PNAS Nexus. 2025 Feb 19;4(2):pgaf028. doi: 10.1093/pnasnexus/pgaf028 (PMC11837328; doi:10.1093/pnasnexus/pgaf028)
Supplement: pgaf028_Supplementary_Data [file pgaf028_supplementary_data.pdf]

Supplementary file 1: Additional figures

Large-scale analysis of fact-checked stories on Twitter reveals graded effects of ambiguity and falsehood on information reappearance

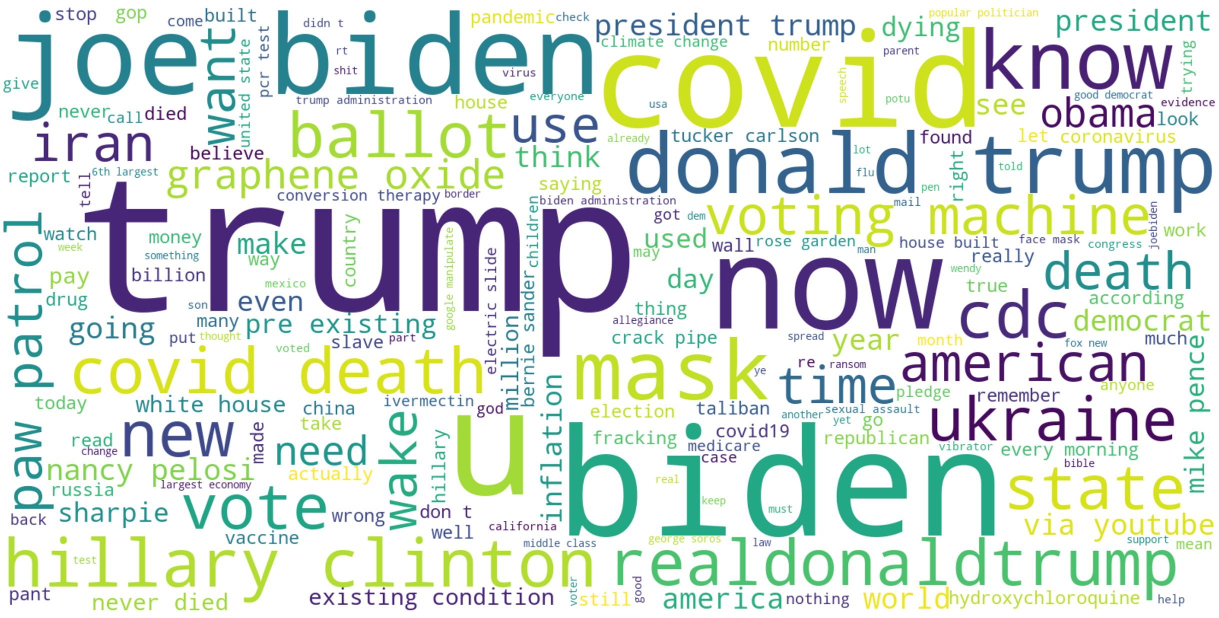

Figure 1: Wordcloud showing the most frequently utilized terms in our tweets. This visualization was created using the Python library wordclouds, employing default settings with a maximum word size constraint. Additionally, we employed a predefined set of stopwords during data preprocessing; the specifics can be found in the corresponding script on OSF.

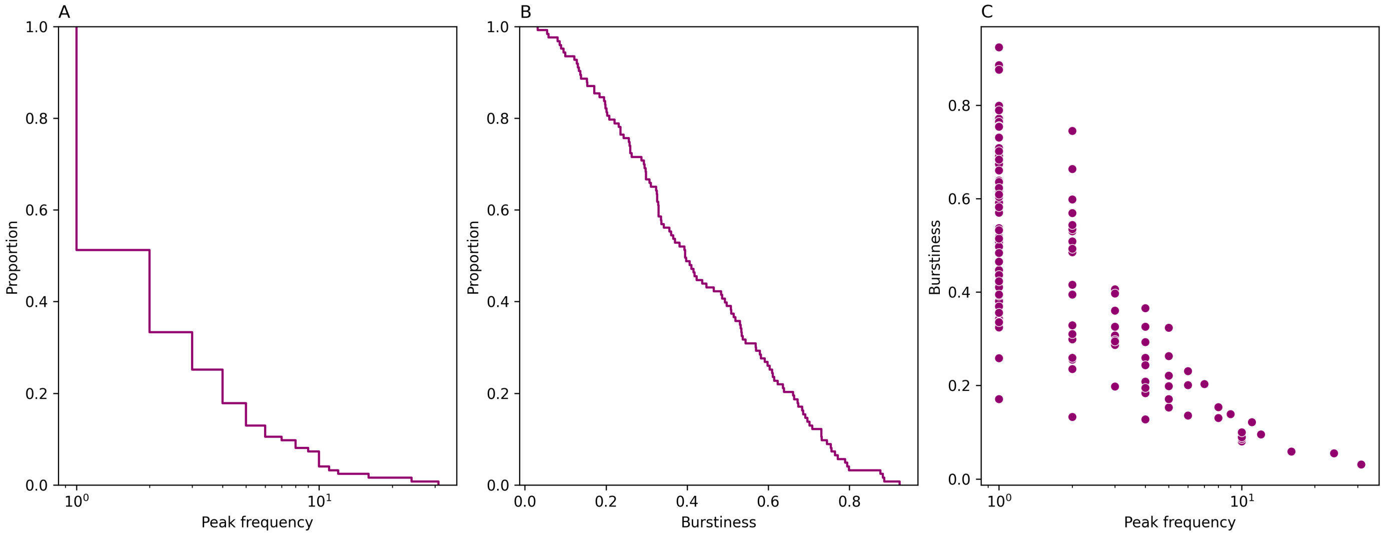

Figure 2: Distributions and correlation of peak frequency and burstiness. Empirical complementary cumulative distribution function (eCCDF) for peak frequency (A) and burstiness (B). The eCCDF reflects the proportion of observations with values above a particular level. (C) Scatter plot of peak frequency and burstiness.

Supplementary file 2: Control analyses

Large-scale analysis of fact-checked stories on Twitter reveals graded effects of ambiguity and falsehood on information reappearance

Poisson models for peak frequency

Zero-truncated negative binomial regression models are designed to handle overdispersion in data by incorporating an additional parameter. In contrast, Poisson regression models do not include this parameter, resulting in a more parsimonious model. Model comparisons confirmed that zero-truncated negative binomial regression models were appropriate for our data. All Poisson models exhibited signs of overdispersion, as evidenced by likelihood ratio tests ($p < 0.001$ for all models), thereby legitimizing the use of zero-truncated negative binomial regressions.

Control models: Cubic and saturated models

To prove the robustness of our superior models (dual effect model for peak frequency and ambiguity model for burstiness), we used two overparametrized models (a cubic and a saturated one) as controls. The cubic model included linear, quadratic, and cubic terms to capture more complex relationships. The saturated model treats the fact-checking rating as a categorical variable, assigning a separate parameter estimate to each category (false, mostly false, mixed, mostly true, true), allowing maximum flexibility in modeling the relationship. This approach ensures that the model captures all possible variation among the categories without imposing any specific structure (like linearity or quadratic relationships).

The superior dual effect model for peak frequency (AIC = 451.95) showed lower AIC values than the overparametrized cubic (AIC = 453.53) and saturated (AIC = 454.9) models, confirming its superior predictive power despite fewer parameters. This was also supported by insignificant likelihood ratio tests (cubic: $\chi^2(1) = 0.42, p = .519$, and saturated: $\chi^2(2) = 1.05, p = .591$).

We found a similar pattern for the superior ambiguity model for burstiness (AIC = −38.13), which showed no meaningful AIC differences to the overparametrized cubic (AIC = −39.14) and saturated (AIC = −37.14) models, legitimizing the use of a more parsimonious model. Insignificant likelihood ratio tests confirmed this result (cubic: $\chi^2(2) = 3.75, p = .154$, and saturated: $\chi^2(3) = 3.75, p = .29$).

One-inflation check for peak frequency

We also checked for one-inflation of the peak frequency, which would indicate an overrepresentation of one counts in the data. We found no evidence of one-inflation in our data, as confirmed by likelihood ratio tests ($p \geq .166$ for all models) performed with the R package ONEINFL (Godwin, 2024), comparing a one-inflation model with a model without one-inflation.

Orthogonalization of linear and quadratic predictors

Predictors within a polynomial regression can be correlated, which may lead to biased estimates of the regression coefficients. To address this, we orthogonalized our polynomials using the R function POLY, which yielded uncorrelated linear and quadratic predictors, allowing us to better isolate both effects. Note that while coefficients cannot be directly interpreted after orthogonalization, p-values still indicate the significance of a predictor.

The orthogonal polynomial regression modeling confirmed the superiority of ambiguity effects over falsehood effects. While still predicting a negative linear effect for peak frequency and a positive linear effect for burstiness, the significance of the linear coefficients declined (peak frequency: $\beta_1 = -3.0, p = .149$, burstiness: $\beta_1 = 0.74, p = .383$), whereas it increased for the quadratic coefficient (peak frequency: $\beta_2 = -5.69, p = 6.58 \cdot 10^{-3}$, burstiness: $\beta_2 = 2.25, p = 9.14 \cdot 10^{-3}$). Notably, in this orthogonal dual effect model for peak frequency, the linear coefficient did not reach significance anymore. These findings confirm the prominence of ambiguity over falsehood effects, and indicate that falsehood effects are less stable than previously reported in the literature.

Text similarity threshold sensitivity analysis

To prove whether the observed effects are not threshold-specific (i.e., they do not only occur at the threshold of .7), we tested our models over a threshold space. We examined a linear threshold space ranging from .5 to .9, incrementing the threshold in steps of .001. This yielded a total of 401 threshold levels (corresponding to the number of tests conducted). The threshold limits were chosen according to Vosoughi et al. (2018), who considered tweets with $S_c \geq .9$ as certainly supportive, while tweets exhibiting $S_c < .5$ were discarded, as they likely do not support the respective story. In general, a S_c threshold of .5 is frequently used in the literature (see, e.g., Vosoughi et al., 2018; Mihalcea et al., 2006) to indicate some degree of similarity.

Peak frequency (Negative binomial regression). In terms of the peak frequency, we adopted a dual effect model in our main manuscript, as given by:

$$\eta_i = \beta_0 + \beta_1 x_i + \beta_2 x_i^2 \tag{1}$$

where η_i is the linear predictor of the response variable, x_i is the fact-checking rating, β_0 is the intercept, and β_1 and β_2 are the linear and quadratic coefficients, respectively.

Regarding the linear coefficient, the effect was reproducible across 94 (23.44%) threshold levels (see Figure 1A), with the largest cluster of adjacent significant thresholds spanning from .622 to .667 (cluster mass:

46 [11.47%]). The median p-value across all threshold levels was .261. This suggests that the linear effect of story credibility on peak frequency is relatively unstable, indicating a rather weak falsehood effect.

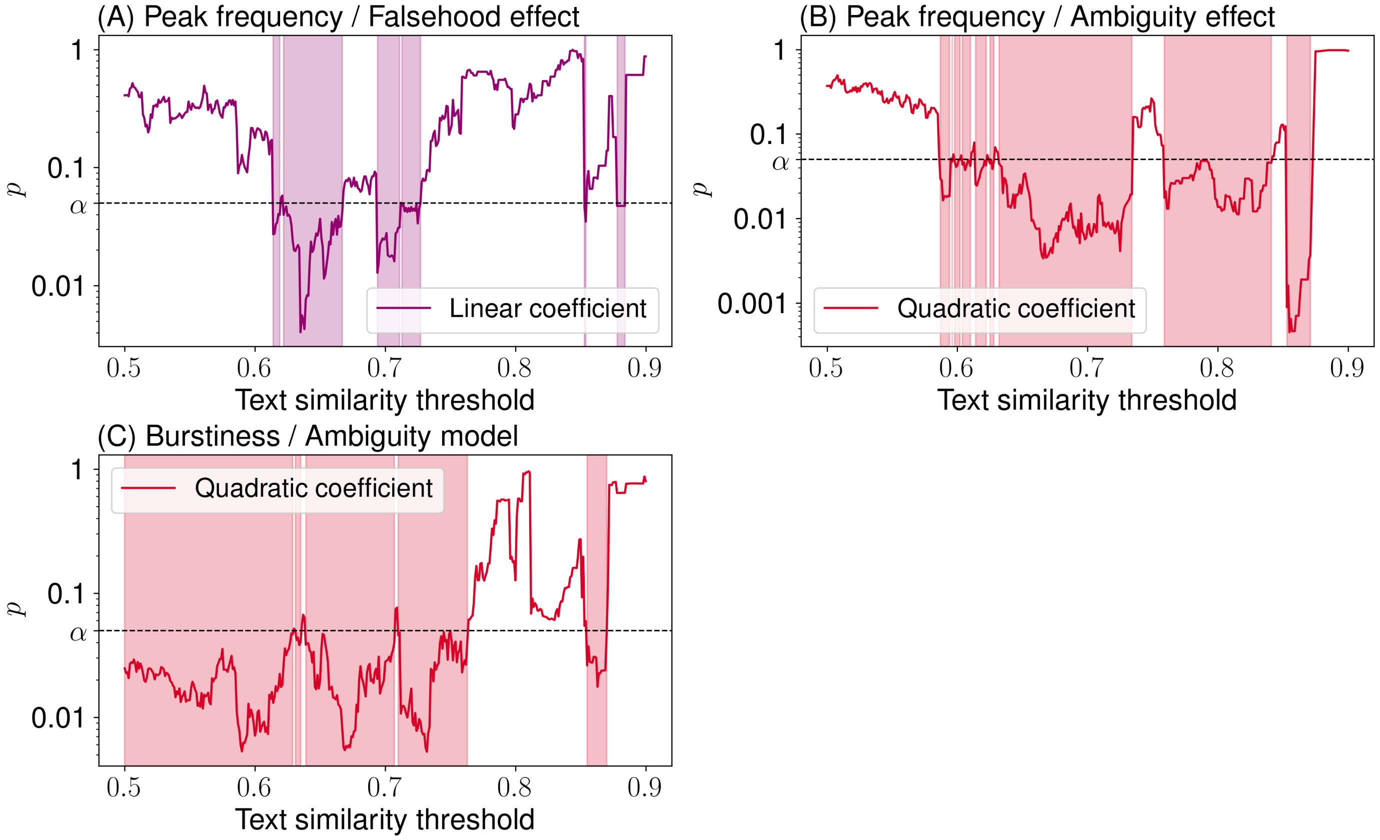

Figure 1: The p-values from the t-tests for coefficient significance across the threshold space (minimum text similarity S_c) are shown for the peak frequency dual effect (A and B) and the burstiness ambiguity model (C). Light-colored regions highlight thresholds where the tests were significant.

For the quadratic coefficient, the effect was reproducible across 239 (59.6%) threshold levels (see Figure 1B), with the largest cluster of adjacent significant thresholds ranging from .632 to .734 (cluster mass: 103 [25.69%]). The median p-value across all thresholds was .034. This suggests a more stable quadratic effect of story credibility on peak frequency, indicating that the ambiguity effect is relatively strong and less sensitive to the chosen threshold.

Burstiness (Beta regression). For burstiness, we found that an ambiguity model explained the data most accurately. This model is given by:

$$\eta_i = \beta_0 + \beta_2 x_i^2 \tag{2}$$

where η_i is again the linear predictor, x_i is the fact-checking rating, β_0 is the intercept, and β_2 is the quadratic coefficient (reflecting the ambiguity effect).

The quadratic coefficient was significantly different from zero across 274 (68.33%) threshold levels (see Figure 1C), with the largest cluster of adjacent significant thresholds ranging from .5 to .629 (cluster mass: 130 [32.42%]). The median p-value across all thresholds was .027. This suggests a stable quadratic effect of story credibility on burstiness, indicating that the ambiguity effect is relatively robust and not heavily dependent on the chosen threshold.

Sensitivity analysis for the minimum number of tweets per story

We conducted a sensitivity analysis to ensure that our findings are robust across different criteria for the minimum number of tweets per story (threshold used in the main analyses: 3000). Specifically, we applied a linear threshold space ranging from 500 to 10000 tweets. If the effect is robust, we would expect relatively large clusters of adjacent significant coefficients across this range. We tested each value within these boundaries, resulting in a total of 9501 threshold levels.

Peak frequency (Negative binomial regression). Regarding the linear coefficient, the effect was reproducible for 1496 (15.75%) threshold levels (see Figure 2A). The largest cluster of adjacent significant threshold levels ranged from 2144 to 3639 (cluster mass: 1496 [15.75%]). The median p-value across all threshold levels was .139. This suggests an unstable linear effect of story credibility on peak frequency, supporting the notion of a rather weak falsehood effect.

In terms of the quadratic coefficient, the effect was reproducible for 7969 (83.88%) threshold levels (see Figure 2B). The largest cluster of adjacent significant threshold levels ranged from 521 to 8485 (cluster mass: 7965 [83.83%]). The median p-value across all threshold levels was $8.67 \cdot 10^{-3}$. This indicates a much more stable quadratic effect of story credibility on peak frequency, suggesting that the ambiguity effect is relatively strong and less dependent on the chosen threshold for the minimal number of tweets per story.

Burstiness (Beta regression). The quadratic coefficient differed significantly from zero for 6859 (72.19%) threshold levels (see Figure 2C). The largest cluster of adjacent significant threshold levels ranged from 2365 to 8485 (cluster mass: 6121 [64.42%]). The median p-value across all threshold levels was .017. This indicates a stable quadratic effect of story credibility on burstiness, suggesting that the ambiguity effect is relatively strong and not heavily influenced by the chosen threshold for the minimal number of tweets per story.

Sensitivity analysis for the peak detection parameters

We employed the peak-finding algorithm proposed by Shin et al. (Shin et al., 2018), including both the minimal height parameter and the minimal distance between peaks. However, the parameter selection could be viewed as arbitrary. To address this, we conducted a control analysis where we systematically varied both parameters. Specifically, the distance between peaks was allowed to vary from 1 to 21 days (step size: 1 day), and the minimal height was varied between 0.01 and 0.5 (step size: 0.01) of the maximum deflection in the time series. This yielded a total of 1,050 parameter combinations.

In line with our previous sensitivity analyses, we observed that the ambiguity effect was considerably more robust than the falsehood effect. For the linear effect of story credibility on peak frequency, we found that it

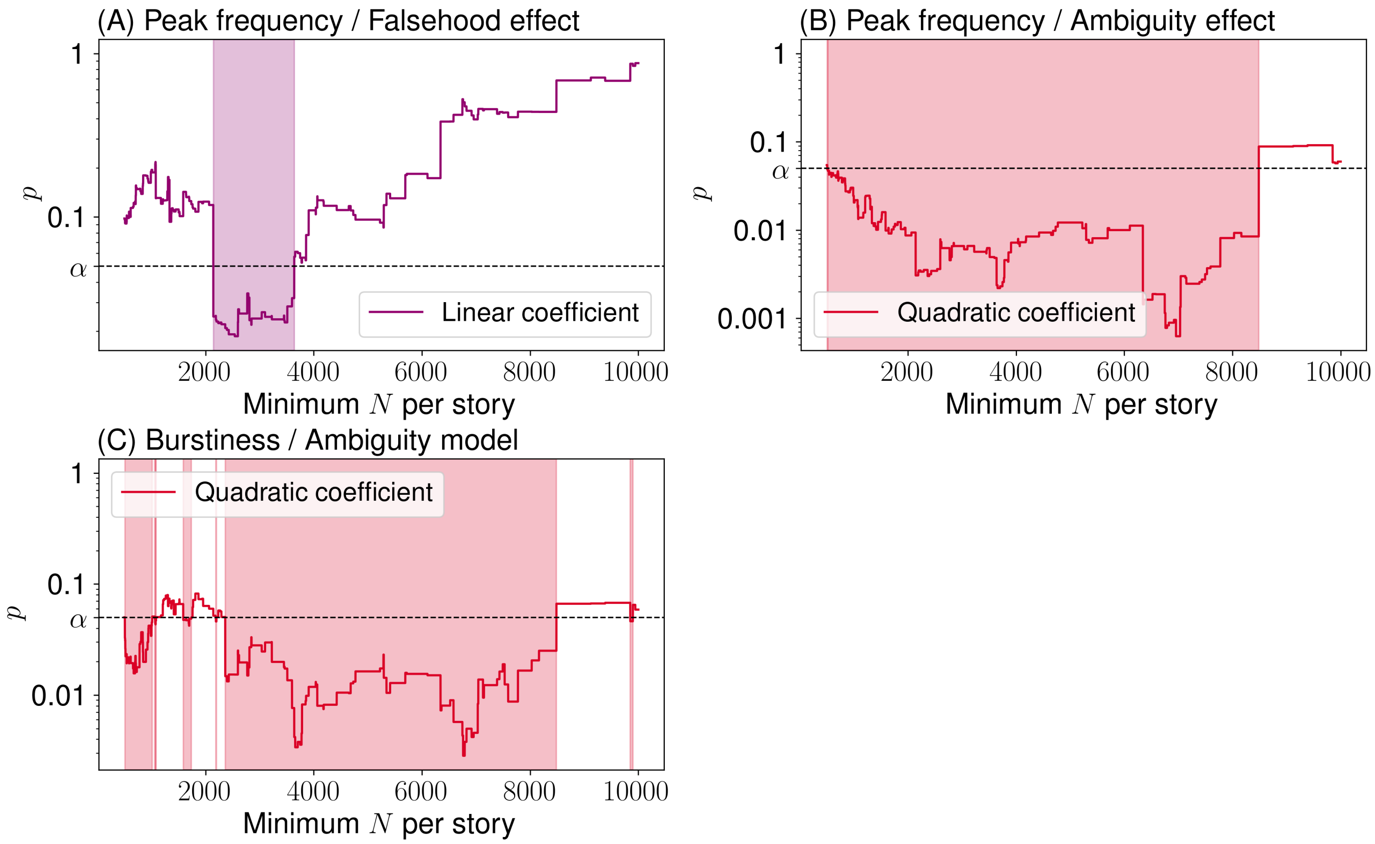

Figure 2: The p-values from the t-tests for coefficient significance across the threshold space (minimum number of tweets per story) are shown for the peak frequency dual effect (A and B) and the burstiness ambiguity model (C). Light-colored regions highlight thresholds where the tests were significant.

was reproducible at $\alpha = .05$ for 258 (24.57%) of parameter combinations (see Figure 3A). At the $\alpha = .01$ level, the linear effect was present for 4 (0.38%) of combinations; however, no combinations reached significance at $\alpha = .001$. The median p-value was .095.

For the quadratic effect, we observed replication at $\alpha = .05$ in 1050 (100.0%) parameter combinations (see Figure 3B). At $\alpha = .01$ and $\alpha = .001$, the effect was present in 719 (68.48%) and 17 (1.62%) combinations, respectively. The median p-value was $6.88 \cdot 10^{-3}$, confirming that the ambiguity effect remains highly robust across the tested parameter space.

Control model incorporating tweet- and user-level covariates

To better account for the effects of covariates, we conducted a control analysis that included a relevant set of covariates. Additionally, we adjusted the resolution level of our analysis from the "story level" to the "peak level," where each peak within a story is represented individually rather than considering the story as a whole. This adjustment enabled a more precise examination of covariate effects on peak frequency. The covariates

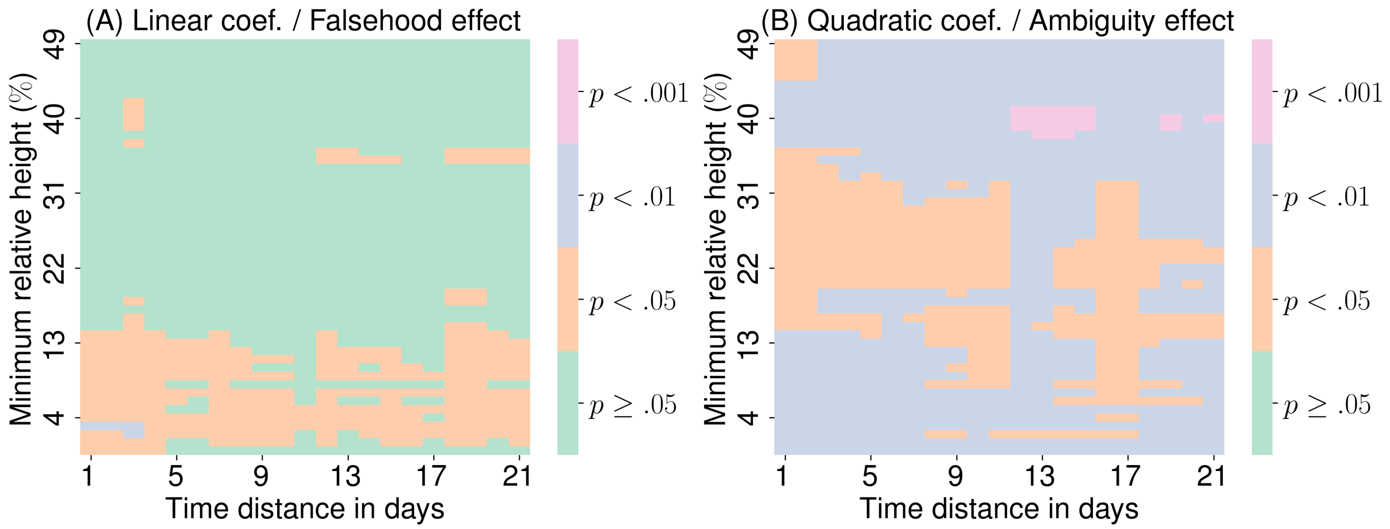

Figure 3: Heatmaps of p-values of the t-tests for coefficient significance (Panel A: Linear coefficient / Falsehood effect, Panel B: Quadratic coefficient / ambiguity effect) across different combinations of the minimal height and distance parameters.

included were:

1. Tweet length
2. Number of hashtags in the tweet
3. Number of mentions in the tweet
4. Text similarity of the tweets with the (original) claim of the story
5. Negative sentiment score (probability of the tweet to be negative; according to TweetNLP; see Camacho-collados et al. (2022))
6. Number of account followers (log_{10}-transformed)
7. Account verified (0 = No, 1 = Yes)

We used a mixed ordinal regression model with the `clmm` function from the `ordinal` package (version: 2023.12-4; see Christensen (2015)) in `R`. The dependent variable was the peak number (1st, 2nd, 3rd, etc.) for each story, with predictor variables including credibility (to capture the falsehood effect), squared credibility (to capture the ambiguity effect), and all previously mentioned control variables. We included a random intercept for each story to account for repeated measures across peaks within the same story, modeling variability between stories. To improve model convergence, we z-transformed the predictor variables. To account for potential alpha-error accumulation, we applied the Benjamini-Hochberg (BH) procedure (Benjamini and Hochberg, 1995), resulting in adjusted p-values (p_{adj}). Table 1 shows the fixed effects of our mixed ordinal regression model.

Table 1: Fixed effects of the mixed ordinal regression model with covariates.

Parameter	Coefficient	SE	CI_low	CI_high	z	p	p_{adj}
β_1 (Falsehood effect)	-0.29	0.209	-0.699	0.119	-1.391	0.164	0.296
β_2 (Ambiguity effect)	-0.626	0.237	-1.09	-0.162	-2.646	0.008	0.045
Number of followers	0.008	0.122	-0.231	0.246	0.064	0.949	0.949
Tweet length	0.051	0.136	-0.215	0.316	0.374	0.709	0.911
Negative sentiment score	0.353	0.137	0.084	0.621	2.576	0.01	0.045
Text similarity	-0.328	0.14	-0.603	-0.054	-2.345	0.019	0.057
Hashtag count	-0.094	0.113	-0.315	0.127	-0.833	0.405	0.607
Mention count	-0.191	0.107	-0.4	0.018	-1.791	0.073	0.165
User verified	-0.023	0.11	-0.238	0.192	-0.209	0.834	0.939

Note. SE = Standard error. CI_low and CI_high correspond the the 95% confidence interval. Please note that predictor variables were z-transformed.

We found that the linear falsehood effect was no longer significant ($\beta_1 = -0.29, p = .164, p_{adj} = .296$), while the significance of the quadratic ambiguity effect remained largely unchanged ($\beta_1 = -0.63, p = 8.14 \cdot 10^{-3}, p_{adj} = .045$), making it the most significant coefficient in the model. This indicates a robust ambiguity effect on story reappearance, while the falsehood effect appears to be more unstable. Additionally, we observed significant effects for text similarity and negative sentiment scores (see Table 1), but only the effect of the negative sentiment scores remained significant after the BH-correction. These results align with the findings of Shin et al. (2018), who noted that stories tend to evolve in their narrative over time (as suggested by the negative coefficient for text similarity) and may become more emotionally charged (reflected in the positive coefficient for negative sentiment scores).

Control analysis excluding "Scam" and "Pants on Fire" categories

Since both the "Scam" and "Pants on Fire" categories may be logically inconsistent with the 5-point Likert scale ranging from "False" to "True" (via "Mixed"), we conducted a control analysis excluding these categories (in total, 9 stories were either "Scam" or "Pants on Fire", corresponding to 114 stories in total).

Peak frequency (Negative binomial regression). For peak frequency, we applied the dual effect model in our main analysis. Excluding these categories confirmed the validity of the dual effect model, with both the linear coefficient ($\beta_1 = -0.33, p = .018$) and the quadratic coefficient remaining significant ($\beta_2 = -0.34, p = .01$). Compared to other models, the dual effect model provided the best fit, as indicated by the lowest AIC values (falsehood: 432.83; ambiguity: 430.5; dual: 427.64).

Burstiness (Beta regression). For burstiness, we adopted the ambiguity model in the main analysis. Excluding the "Scam" and "Pants on Fire" categories confirmed the ambiguity model, with the quadratic coefficient remaining significant ($\beta_2 = 0.11, p = .04$). The ambiguity model also exhibited the best fit, as shown by the AIC values (falsehood: -31.09; ambiguity: -34.14; dual: -35.23).

Reanalysis of Mitra et al. (2017)

We conducted a preliminary reanalysis of the data provided by Mitra et al. (2017); Mitra and Gilbert (2015) to determine whether a linear, quadratic, or dual effect model better predicts their data. The dataset comprises 1378 events, of which 1157 had at least 3000 tweets; only these events were included in our analysis. Their rating scale, similar to ours, ranges from -2 to 2 (-2: Certainly Accurate, -1: Probably Accurate, 0: Uncertain, 1: Probably Inaccurate, 2: Certainly Inaccurate). Each event was rated by 30 independent raters, and we used the mean credibility rating as the independent variable. Notably, almost all events (except one outlier, which was excluded from the analysis) were rated as uncertain, probably accurate, or certainly accurate (see Figure 4), limiting the dataset's generalizability. We focused on burstiness since the events were rather short, with fewer than 50 having two peaks at least 7 days apart. Over all events, burstiness ranged from 0.15 to 1.0 with a mean of 0.73 (SD: 0.21) and a median of 0.76 (IQR: $0.58 - 0.92$). The distribution of burstiness was only moderately skewed (skewness: -0.51). Thus, burstiness was higher in their dataset as in our dataset, probably due to the fact they collected data of events rather than stories.

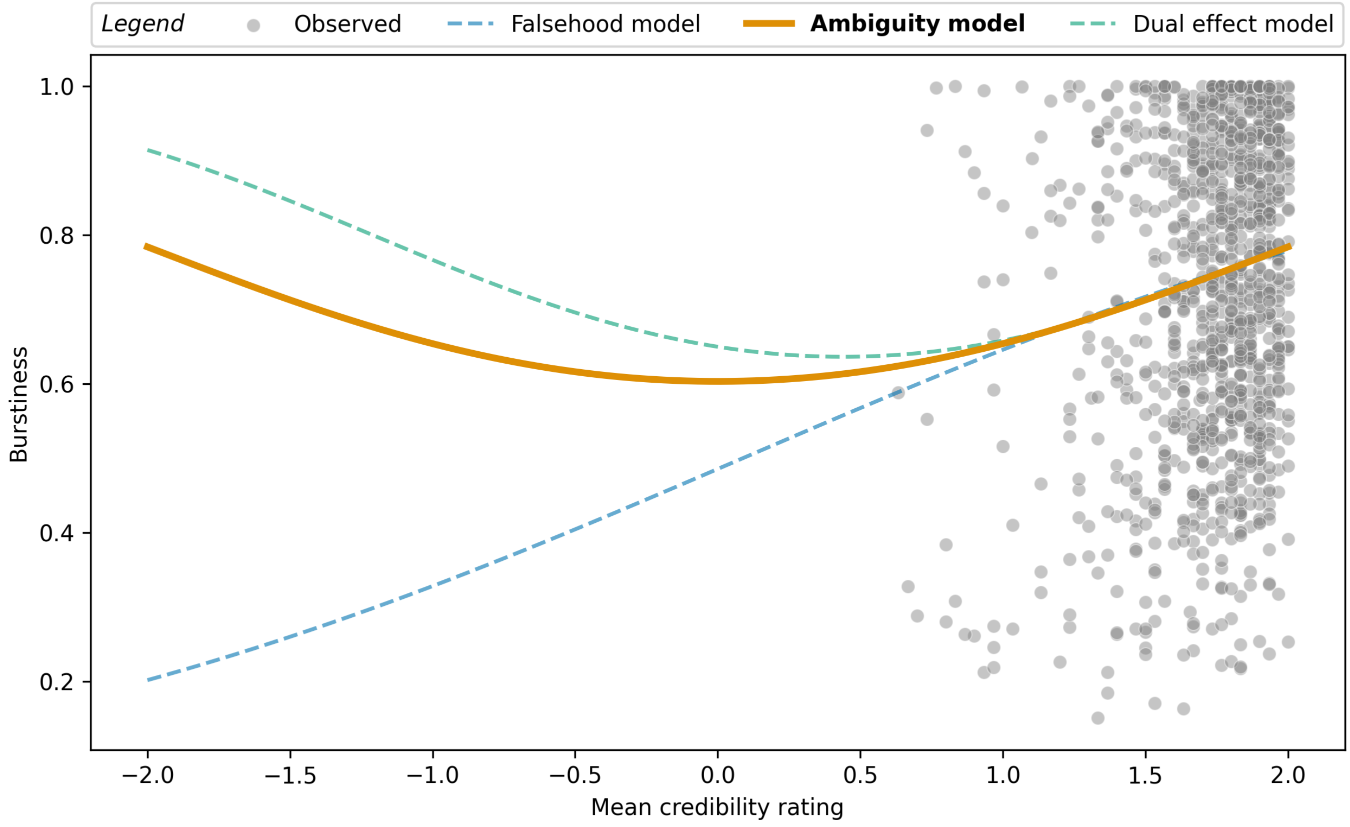

Figure 4: Falsehood, ambiguity, and dual effect models for burstiness. Relationship between rating and burstiness, where dashed curves represent the fits of the inferior models, and the solid line represents the fit of the superior model.

We evaluated null, falsehood, ambiguity, and dual effect models. The main results are presented in Table 2. Consistent with our primary findings, the ambiguity model best fit the data, evidenced by a lower AIC (AIC = -1010.76) compared to the falsehood (AIC = -1010.09) and dual effect models (AIC = -1008.82). However, the difference between the ambiguity and falsehood models was small (ΔAIC = 0.67), indicating only a slightly higher likelihood for the ambiguity model (it was 1.4 times as likely as the falsehood model). This small difference might be due to the absence of events rated as inaccurate in the dataset, allowing only half of the curve to be modeled. Nevertheless, the slight superiority of the ambiguity model (see also Figure 4), despite these limitations, suggests that ambiguity plays a more significant role in the reappearance of information.

Table 2: Main results of the beta regression models for burstiness.

	Coefficient			Model diagnostic			Relative likelihood			
Model	β_0	β_1	β_2	R^2_{McF}	$\log(L)$	AIC	Null	Falseh.	Ambig.	Dual eff.
Null	1.08***	–	–	–	496.42	-988.84	–	$2.43 \cdot 10^{-5}$***	$1.74 \cdot 10^{-5}$***	$4.6 \cdot 10^{-5}$***
Falsehood	-0.06	0.66***	–	.016	508.04	-1010.09	41,094.27***	–	0.72	1.89
**Ambiguity**	0.42**	–	0.22***	.016	508.38	-1010.76	57,358.69***	1.4	–	2.64
Dual effect	0.62	-0.27	0.3	.016	508.41	-1008.82	21,733.94***	0.53	0.38	–

Note. *: $p < 0.05$, **: $p < 0.01$, ***: $p < 0.001$. R^2_{McF} reflect pseudo-R^2 according to McFadden. Asterisks behind the relative likelihoods reflect results of the likelihood ratio tests (only applicable if the number of model parameters differ).

Generalizability of results across fact-checking sources

To evaluate the robustness and applicability of our results across different subdatasets, we tested our main models (the dual-effect model for peak frequency and the ambiguity model for burstiness) separately for the two fact-checking sources, Snopes and PolitiFact. We anticipated that the model predictions for both sources would exhibit similar trends, specifically showing evidence of both an ambiguity effect and a falsehood effect. Such findings would suggest a degree of generalizability, as Snopes and PolitiFact arguably differ in their story selection criteria and methods of analysis.

As expected, we found broadly similar patterns for both sources, although some notable differences emerged. For the peak frequency model, both sources exhibited a negative linear trend, with the effect estimate being stronger for PolitiFact ($\beta_1 = -0.37, p = .246$) than for Snopes ($\beta_1 = -0.12, p = .486$). The quadratic coefficients were more comparable, with PolitiFact ($\beta_2 = -0.38, p = .103$) showing only a slight advantage over Snopes ($\beta_2 = -0.31, p = .072$), as illustrated in Figure 5A.

For the burstiness model, the quadratic effect estimates also showed minimal differences (PolitiFact: $\beta_2 = 0.12, p = .097$; Snopes: $\beta_2 = 0.09, p = .199$). However, visual inspection showed that stories fact-checked by PolitiFact tended to reappear more frequently, resulting in lower burstiness compared to Snopes. This may reflect differing selection criteria, with PolitiFact's focus on political content likely garnering more public attention on Twitter, a platform known for its political discourse.

This analysis should be considered exploratory due to the reduced statistical power when analyzing the sources separately. Consequently, we report trends rather than definitive statistical patterns. Nonetheless, these trends align with previous research, such as Shin et al. (2018), which suggested that false political

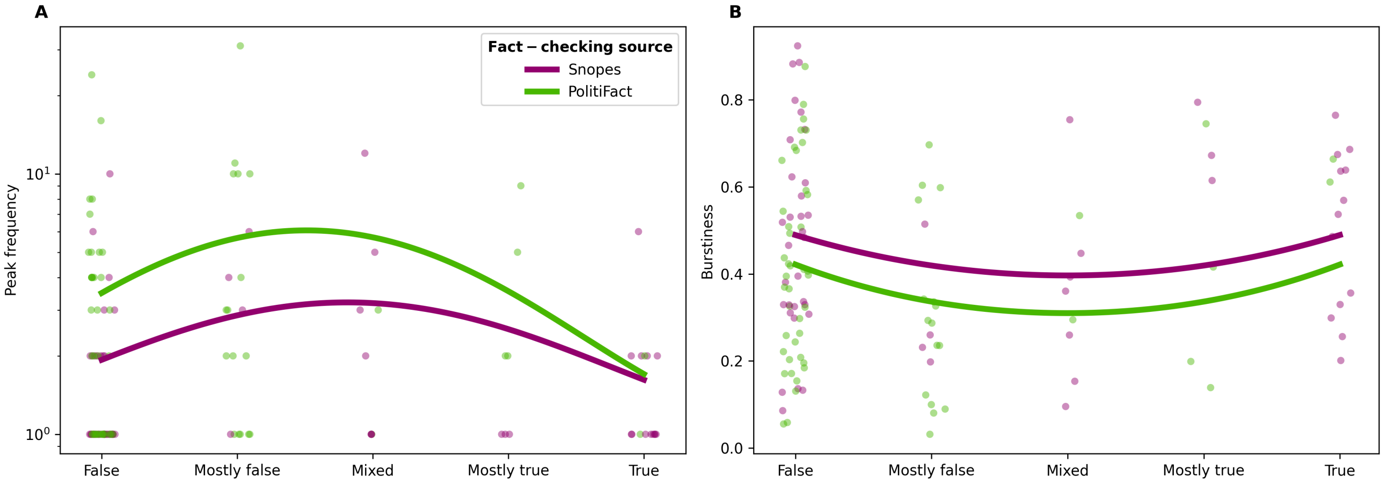

Figure 5: Model predictions for the response variables, analyzed separately for each fact-checking source. (A) Dual-effect model predictions for peak frequency across Snopes and PolitiFact. (B) Ambiguity model predictions for burstiness across both sources.

content may exhibit a stronger falsehood effect. Future studies should further investigate the static and procedural differences between fact-checking sources. Our findings provide initial evidence that PolitiFact's focus on political stories may influence the dynamics of content reappearance.

Temporal stability of false stories: Exploring potential effects of Twitter policy changes

To better understand potential biases in our main response variables over time, we conducted an additional analysis to examine whether the peak frequency or burstiness of false stories exhibited any temporal trends or irregularities. Such patterns may reflect shifts in Twitter's moderation policies, as certain types of misinformation might be suppressed or altered in their reappearance frequency following policy changes. This effect was particularly evident after major political events, such as the 2016 U.S. presidential election or the attack on the United States Capitol on January 6, 2021 (see University of Pittsburgh Center for Social and Urban Research, 2024; McCabe et al., 2024).

We examined whether any irregularities occurred over time in our response variables for false stories, hypothesizing that any policy changes affecting our data would manifest as relatively sudden changes in these variables. To investigate this, we conducted two complementary analyses.

First, we divided our data into an early and a late half using a temporal median split. This comparison provided an overall view of how the peak frequency changed over time, effectively highlighting broader trends while canceling out smaller fluctuations in the time course. This approach offered a straightforward way to detect substantial temporal shifts in the data.

However, recognizing that the median split might not be sensitive enough to detect abrupt or temporary policy-driven changes, we also conducted a more detailed analysis by treating time as a continuous variable.

In this approach, we expected to observe non-linear patterns in the data over time if policy changes had influenced our response variables.

To capture these potential nonlinearities, we employed generalized additive models (GAMs). GAMs are a flexible statistical method that determines the relationship between a response variable and a predictor variable without requiring a predefined assumption about the shape of the relationship. This flexibility is achieved by modeling the relationship as a smooth function, such as a spline. Crucially, GAMs account for model complexity by penalizing overly complex models with excessive parameters, ensuring a balance between fit and interpretability. As a result, GAMs are particularly effective at detecting significant non-linear patterns in data. We used the R library MGCV (version: 1.9-1) to fit the GAM model (smooth term were applied to the the time passed in days since the first story using cubic splines). For simplicity and interpretability, we log_{10}-transformed the peak frequency before computing the GAMs. This transformation stabilized variance and facilitated clearer insights into potential temporal irregularities. We report the smooth terms by presenting their effective degrees of freedom (EDF) and significance levels. The EDF quantifies the complexity of the smoothing term: an EDF close to 1 indicates a linear relationship, while higher values (e.g., ≥ 2) suggest increasing degrees of non-linearity.

We found no meaningful differences for peak frequency between early and late stories, as confirmed by a zero-truncated negative binomial regression ($\beta_t = 0.064, z = 0.175, p = .861$; see Figure 6A). The same was found for burstiness ($\beta_t = 0.1334, z = 0.849, p = .396$; see Figure 6C). We also found no evidence that our response variables were driven by any non-linear processes: The smooth term of the GAM models were insignificant for both peak frequency (EDF $= 1.078, p = .521$; see Figure 6B) and burstiness (EDF $= 1.217, p = .571$; see Figure 6D). Both the low EDFs and the insignificance of both smoothing terms indicate that there are no detectable linear or nonlinear effects of time on our response variables.

In summary, we found no evidence that the response variables of our false stories were influenced by story onset time in any meaningful way. However, this finding should be interpreted with caution and may only provide initial evidence that our data was minimally or moderately affected by Twitter policy changes. For future studies, using a dataset with a larger number of stories could improve the ability to detect subtle effects of policy changes on the outcome variables.

Validating model significance with permutation tests

To assess the significance of our models, we performed permutation tests. The key advantage of permutation tests is that they compare observed model statistics (e.g.,z- or t-values) against empirical null distributions generated by resampling the data. This approach is more robust to violations of model assumptions (e.g., normality or homoscedasticity), making it particularly suitable for smaller datasets or non-standard conditions.

For simplicity and feasibility, we focused on recomputing the significance of each coefficient in our main models: the dual-effect model for peak frequency and the ambiguity model for burstiness. We used the z-values of the coefficients as our test statistic, comparing the observed z-values to null distributions obtained by randomly permuting the response variable while keeping the predictors fixed. A total of $n = 10000$ permutations was conducted, providing sufficient precision to estimate p-values empirically.

The permutation-derived p-values ($\hat{p}$) confirmed the ambiguity effect, though the significance of the falsehood effect was weakened. For peak frequency, the quadratic ambiguity effect remained significant ($p = .007$ vs. $\hat{p} = .02$), but the linear falsehood effect lost significance ($p = .023$ vs. $\hat{p} = .06$). For burstiness, the

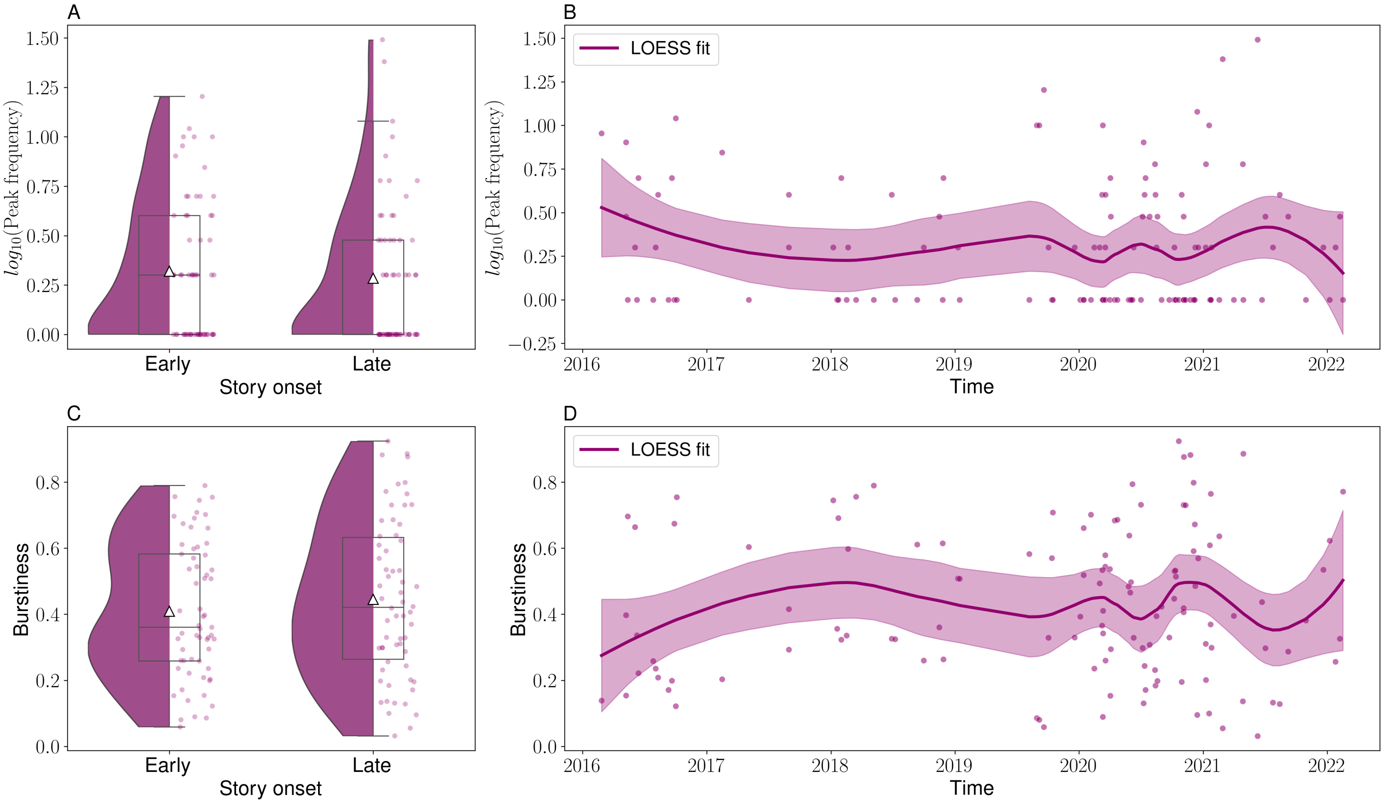

Figure 6: Time dependency of the peak frequency for false stories. (A, C) No differences in the peak frequency (A) and burstiness (C) after a median split of time. Colored areas and white triangles reflect kernel density estimations (KDEs) and means, respectively. (B, D) No linear or non-linear modulation of peak frequency (B) and burstiness (D) over time. The solid pink curve reflects a locally estimated scatterplot smoothing (LOESS) fit (smoothing span: .5); error bars show 95% confidence intervals.

significance of the ambiguity model remained nearly unchanged ($p = .029$ vs. $\hat{p} = .03$).

In summary, the ambiguity effect was robust across both response variables, remaining significant under permutation testing. However, the falsehood effect was less stable, with its significance diminished when tested against the empirical null distribution. This highlights the utility of permutation tests in verifying the robustness of statistical results, particularly for effects that may be sensitive to model assumptions.

References

Benjamini Y and Hochberg Y (1995) Controlling the False Discovery Rate: A Practical and Powerful Approach to Multiple Testing. *Journal of the Royal Statistical Society Series B: Statistical Methodology* 57(1). DOI: 10.1111/j.2517-6161.1995.tb02031.x.

Camacho-collados J, Rezaee K, Riahi T, Ushio A, Loureiro D, Antypas D, Boisson J, Espinosa Anke L, Liu F and Martinez Cámara E (2022) TweetNLP: Cutting-Edge Natural Language Processing for Social Media. In: *Proceedings of the 2022 Conference on Empirical Methods in Natural Language Processing: System Demonstrations*. Abu Dhabi, UAE: Association for Computational Linguistics, pp. 38–49. DOI: 10.48550/arXiv.2206.14774.

Christensen R (2015) Package Ordinal: Regression Models for Ordinal Data. *R package version 2015* .

Godwin RT (2024) One-inflated zero-truncated count regression models [Preprint] DOI:arXiv:2402.02272v1.

McCabe SD, Ferrari D, Green J, Lazer DMJ and Esterling KM (2024) Post-January 6th deplatforming reduced the reach of misinformation on Twitter. *Nature* 630(8015): 132–140. DOI:10.1038/s41586-024-07524-8. URL `https://doi.org/10.1038/s41586-024-07524-8`.

Mihalcea R, Corley C and Strapparava C (2006) Corpus-based and knowledge-based measures of text semantic similarity. In: *Proceedings of the National Conference on Artificial Intelligence*, volume 1. Boston, Massachusetts: AAAI Press, pp. 775–780.

Mitra T and Gilbert E (2015) CREDBANK: A large-scale social media corpus with associated credibility annotations. In: *Proceedings of the 9th International Conference on Web and Social Media, ICWSM 2015*. pp. 258–267. DOI:10.1609/icwsm.v9i1.14625.

Mitra T, Wright G and Gilbert E (2017) Credibility and the dynamics of collective attention. In: *Proceedings of the ACM on Human-Computer Interaction*, volume 1. New York, NY, USA: Association for Computing Machinery, pp. 1–17. DOI:10.1145/3134715.

Shin J, Jian L, Driscoll K and Bar F (2018) The diffusion of misinformation on social media: Temporal pattern, message, and source. *Computers in Human Behavior* 83: 278–287. DOI:10.1016/j.chb.2018.02.008.

University of Pittsburgh Center for Social and Urban Research (2024) Social Media Election Policy Tracker. URL `https://apps.cyber.pitt.edu/social-media-election-policies`.

Vosoughi S, Roy D and Aral S (2018) The spread of true and false news online. *Science* 359(6380): 1146–1151. DOI:10.1126/science.aap9559.
